# Personality and the physician-patient relationship as predictors of quality of life of cardiac patients after rehabilitation

**DOI:** 10.1186/1477-7525-8-100

**Published:** 2010-09-14

**Authors:** Erik Farin, Milena Meder

**Affiliations:** 1 University Medical Center Freiburg, Dept. of Quality Management and Social Medicine, Breisacher Str. 62/Haus 4 D-79106, Freiburg, Germany

## Abstract

**Background:**

Numerous studies document the influence of psychosocial variables on the course of coronary heart disease. This study examines the influence of personality traits (trait anger, cynicism) and aspects of the physician-patient relationship (promoting patient participation by the physician, active communication behavior of the patient, trust in the physician) on the health related quality of life (HRQOL) of cardiac patients after rehabilitation.

**Methods:**

N = 331 patients with chronic ischemic heart disease were surveyed using questionnaires at two time points (beginning and end of 3-weeks inpatient rehabilitation). In addition, characteristics of the disease and cardiac risk factors were provided by the physician. HRQOL was measured using a total of six scales and three instruments: SF-12, MacNew questionnaire, and SAQ. Hierarchical regression analyses were carried out to predict HRQOL after rehabilitation, in which the baseline values of HRQOL, sociodemographic variables, characteristics of the disease and risk factors, personality traits, and finally the aspects of the physician-patient relationship were included stepwise. As a number of variables were used for the regression models, multiple imputation was conducted.

**Results:**

The baseline values explain most of the variance (42%-60%). After controlling the baseline values, the sociodemographic variables explain up to 5% incremental variance of HRQOL, with income being the most important predictor. The characteristics of the disease and cardiac risk factors explain between 0.4% and 3.8% incremental variance, however, variance increase is often not significant. The personality traits added in the fourth step explain up to 2% additional variance; trait anger is a significant predictor of HRQOL in three of the six scales. The features of the physician-patient relationship included in the last step lead to a significant increase in explained variance (between 1.3% and 3.9%) for all six scales. In particular, the physician's promotion of patient participation has a significant influence. The overall explanation of variance for HRQOL is between 50% and 64%.

**Conclusions:**

Low income, a high level of trait anger, and low patient participation are significant risk factors, even if a number of potential confounders are adjusted. Research is needed that shows which causal pathway low income functions on and what therapies in rehabilitation can mitigate the disadvantage of persons with a high level of trait anger. The providers should implement measures to actively integrate rehabilitation patients in treatment (e.g. encourage them to ask questions).

## Introduction

There is extensive empirical evidence that psychological and psychosocial characteristics have an influence on the incidence and prognosis of coronary heart disease (CHD) (e.g. [1,2]). Additionally, the influence of psychosocial variables on risk factors for CHD such as hypertension, smoking behavior, serum cholesterol, and body mass index has been shown [3-6]. Among the psychological characteristics most frequently discussed in this context are the personality characteristics anger and cynicism.

With reference to Spielberger's State-Trait Anger Expression Inventory (STAXI) [7] and the concept of anger on which this instrument is based, a differentiation is frequently made between trait anger, anger expression, and anger suppression. Trait anger is considered a personality trait characterized by the fact that the person perceives many situations as annoying and thus experiences frequent, intense anger [8]. Studies that have examined the influence of trait anger come to the conclusion that even after adjusting standard risk factors such as age, race, body mass index, education, and smoking, a correlation with carotid artery atherosclerosis [9,10] or high blood pressure [3] can be proven.

Cynicism can be considered the cognitive component of the multidimensional construct "hostility" [11,12]. The construct includes negative beliefs about human nature and the belief that others are potentially threatening antagonists, who frequently have negative intentions and should thus be met with caution and distrust. Anger and cynicism are viewed as independent risk factors for CHD, however, they are related, as anger can also be considered an affective component of hostility [11,13]. Studies have shown that cynicism or closely related concepts such as hostile attributions are predictors of mortality in patients with CHD [14] and are associated with a higher probability of the occurrence of adverse events (e.g. hospitalization for angina, nonfatal myocardial infarction) [15]. In the normal population as well, cynicism appears to be associated with an increased risk of incident myocardial infarction (cf. [16]), however, there are also studies that find no correlation after adjusting for relevant risk factors [17]. Many studies examine either anger or cynicism; some (such as [18]) analyze the influence of both variables in parallel.

When examining the relevance of anger and cynicism, the primary focus was on mortality, the incidence of new cardiac diseases, or the occurrence of impaired body functions. Health-related quality of life (HRQOL) or other patient-reported outcomes were given less consideration, although improving or maintaining HRQOL is very important for chronic cardiac patients and - particularly in the rehabilitation phase - is an important treatment goal (cf. [19,20]). Julkunen and Ahlström [21] found in a large sample of patients with an increased risk of cardiovascular disease that cynicism and anger correlate with HRQOL. The impact of cynicism and anger was, to a great extent, mediated through sense of coherence. A few sociodemographic variables were considered as confounding factors, but not body function parameters or other cardiac risk factors. In a series of studies [22,23], Shen et al. developed prognosis models for predicting the HRQOL of cardiac patients following rehabilitation and they come to the conclusion that hostility affects HRQOL directly and indirectly through depression. However, in the two studies mentioned, anger was not taken into consideration.

Since the personality variables anger and cynicism (especially the latter) have a direct effect on the form of social interaction, it can be expected that they have a correlation to the quality of the physician-patient relationship. However, we are not aware of any study that has examined both the influence of these personality variables and the influence of the physician-patient relationship on the HRQOL of CHD patients after rehabilitation. Of the studies mentioned above, only the studies by Shen et al. [22,23] study rehabilitation care; the authors examine patients in outpatient rehabilitation.

While research groups that have dealt with the influence of anger and cynicism on cardiac disease are frequently based in behavioral medicine or psychosomatic medicine, there are studies on the significance of physician-patient communication from the field of provider-patient research. These studies show that aspects of the physician-patient relationship such as involving the patient in decision-making processes [24,25], positive assessment of communication and interaction by the patient [26,27], and patient's trust in the physician [28] affect not only adherence, but the patient-reported outcome of treatment as well.

The objective of our study is to address elements of these two research traditions in parallel. We examine two hypotheses:

1. Even after adjusting for relevant sociodemographic characteristics, characteristics of the CHD (including risk factors), and the HRQOL at the beginning of inpatient rehabilitation, the personality traits cynicism and anger correlate with the HRQOL after rehabilitation.

2. Even after adjusting for relevant *sociodemographic characteristics, characteristics of the CHD (including risk factors*), the HRQOL at the beginning of inpatient rehabilitation, and the personality traits *cynicism *und *anger*, aspects of the *physicianpatient relationship *perceived by the patient correlate with the HRQOL after rehabilitation.

Through statistical controlling for HRQOL at the beginning of rehabilitation, it is examined whether the personality traits and the physician-patient relationship allow the improvements in the HRQOL that can be achieved by inpatient rehabilitation to be predicted. According to the findings available in literature on confounding influence factors for CHD patients (e.g. [29-33]) age, gender, living with a partner, education, employment, and income will be regarded as sociodemographic confounders. The existence of an acute event and if applicable, the kind of surgery performed before rehabilitation (PTCA, bypass) are important characteristics of the CHD. In addition, various body function parameters and risk factors will be controlled (body mass index (BMI), smoker, total cholesterol, LDL cholesterol, systolic blood pressure, diastolic blood pressure, and the risk score described by Daly [34]). Patient participation in the sense of shared decision making (SDM) [35] and trust in the physician are considered aspects of the physician-patient relationship.

## Methods

### Sample

The study has been approved by the ethics committee of the University Freiburg (approval number 63/08). Patients with chronic ischemic heart disease who were undergoing inpatient rehabilitation were surveyed. In Germany, inpatient cardiac rehabilitation aims at achieving the best possible regeneration of the patient's cardiac capacities with respect to all psychosocial aspects in order to re-integrate the patient into social and working life [36]. The goals of inpatient rehabilitation are similar to those of outpatient rehabilitation, but patients in inpatient rehabilitation are likely to be older, less mobile, and less often employed [36]. Cardiac rehabilitation is a multidisciplinary activity. Besides physicians, the rehabilitation team includes psychologists, nursing staff, exercise therapists, physiotherapists, nutrionists and social workers. The patient generally has 4-5 therapy sessions a day on workdays. Rounds and consultations with the physician take place at least once a week, with additional consultations by appointment. Normally, there is a physician responsible for the patient who sees him frequently during the hospitalization period. Depending on the situation and extent of medical care required, the patient may have contact with other physicians.

The patient questionnaires were given only to patients who were able and willing to fill out the questionnaires (informed consent). N = 742 patients were asked to participate, N = 331 agreed. The percentage of patients that did not fill out the questionnaire (decliners) was 55.4%. The most important reason for non-inclusion was refusal to participate (46.4%), followed by cognitive or physical limitations (13.0%) and language problems (6.5%). For 34.2% of the patients no reason for noninclusion was reported. Table 1 provides information on the patients in the study.

**Table 1:** Respondent characteristics

Average age (SD)	61.1 (10.9)
**Gender**	
% of men	82.4

**Level of education (highest level Completed)**	
% elementary school	52.6

**Employment**	
% employed	41.0

**Monthly household income (%)**	
< 500 Euro	4.0
500 -1.000 Euro	10.0
1.000 - 1.500 Euro	23.3
1.500 - 2.000 Euro	24.1
2.000 - 2.500 Euro	19.3
2.500 - 3.000 Euro	7.2
3.000 - 3.500 Euro	5.2
> 3.500 Euro	6.8

**Acute event/surgery**	
% Myocardial infarction	65.7
% PTCA	62.3
% Bypass (Multiple choices possible)	34.7

**Chronification (%)**	
Acute event	45.7
< 1 year	29.0
1-2 years	6.1
3-5 years	6.5
6-10 years	4.1
> 10 years	7.2

### Instruments

At the beginning and end of the inpatient rehabilitation lasting an average of 21 (+/-3.1) days, the patients were asked to fill out a questionnaire that, in addition to sociodemographic information, included the SF-12 [37] (scales: physical component PC and mental component MC), MacNew [38] (scales: physical limitation PL, emotional functioning EF, social functioning SF), Seattle Angina Questionnaire SAQ [39] (scale considered here: physical limitation PL), Perceived Involvement in Care Scales PICS [40] (scales: doctor facilitation DF, patient information PI), Trust in Physician [41], MMPI-2 scale cynicism [42], and STAXI scale trait anger [7]. Several instruments for quality of life were implemented in parallel to enable statements about the method-related variance. The rehabilitation physicians filled out a documentation sheet at the beginning and end of rehabilitation that covered mainly characteristics of the disease and cardiac risk factors.

For the study reported here, the respective German language versions were used, which are available for all instruments and whose psychometric quality criteria have already been tested in various studies and were found to be satisfactory to good overall (cf. e.g. [43,44] for the HRQOL instruments).

### Analyses

#### Multiple imputation

Since we conduct regression analyses with a large number of predictors that all have a certain percentage of missing values, a method using casewise deletion would have many disadvantages (cf. [45]). Therefore, multiple imputation [46] was used. First, all persons with more than 50% missing values (cf. [45]) were eliminated from the data set. This reduced the data set to N = 319 cases. Then five imputed data sets were created using NORM software [47] according to the recommendations of Rubin [46]. An expectation-maximization algorithm and the data augmentation procedure integrated in the NORM software were applied. We examine the convergence behavior with NORM's plotting procedure. The plots show rapidy coverging series and low autocorrelation. The analyses presented in the results section were carried out with all five imputed data sets. The relevant parameters (regression coefficients, standard errors, etc.) were combined according to the rules presented by Rubin [46].

#### Regression analyses

Hierarchical regression analyses were performed, in which first the baseline values with respect to the respective dependent variables (HRQOL scales) were included. In the second block, all confounding sociodemographic variables that were listed in the introduction were added, in the third step characteristics of the CHD, body function parameters, and risk factors, and in the fourth block, the personality variables (cynicism and anger). Finally, in the fifth step, the characteristics of physician-patient relationship perceived by the patient were included. A stepwise method of variable inclusion (PIN = .05 POUT = .10) was employed. The predictors that were included in the model in at least two of the five imputed data sets were considered as potentially relevant predictors. With this restriction, more sparse models could be specified and problems of multicollinearity avoided. A separate model was specified for each of the six dependent variables (two SF-12 scales, three MacNew scales, one SAQ scale). Finally, the regression models that consisted only of potentially relevant predictors were again applied to all five imputed sets. Here again, hierarchical regression analyses were computed, but now using the forced entry method of variable inclusion, making the results in the five imputed data sets directly comparable. For the diagnosis of multicollinearity, the variance inflation factor (VIF) was calculated. Values over 5 can be considered as an indication of multicollinearity [48]. Statistical analyses were performed using SPSS 17.0 [49].

## Results

Table 2 shows descriptive statistics for the HRQOL scales, the personality variables and the characteristics of physician-patient relationship. For the HRQOL scales, the table also shows the effect sizes of the change after rehabilitation (difference of the mean values divided by the pretest standard deviation); the values are .22 to .57, in the range of low to moderate effects (cf. [50]).

**Table 2:** Descriptive statistics

	Minimum in dataset	Maximum in dataset	Mean	Standard deviation	Effect size (for HRQOL scales)
**HRQOL**					

SF-12: PC					
Start of rehabilitation	8,47	58,51	34,09	10,29	.32
End of rehabilitation	7,51	65,11	37,34	10,87	

MacNew: PL					
Start of rehabilitation	1.60	7	4.69	1.22	.46
End of rehabilitation	1,99	7	5,25	1,10	

SAQ: PL					
Start of rehabilitation	0	100	56,35	25,29	.22
End of rehabilitation	0	100	61,85	24,78	

SF-12: MC					
Start of rehabilitation	9,84	70,14	46,39	12,18	.39
End of rehabilitation	11,81	75,37	51,09	11,60	

MacNew: EF					
Start of rehabilitation	1,17	7	4,79	1,24	.47
End of rehabilitation	1,33	7	5,37	1,13	

MacNew: SF					
Start of rehabilitation	1,18	7	4,56	1,33	.57
End of rehabilitation	1,80	7	5,32	1,14	

**Personality variables**					

Cynicism	0	23	12,63	5,17	

Trait anger	10	37	18,24	5,01	

**Physician patient relationship**					

PICS: DF	1	4	2,94	0,72	

PICS: PI	1	4	3,01	0,75	

Trust in physician	25,90	100	79,58	14,44	

Personality variables were measured at the beginning of rehabilitation, variables of the physician-patient relationship at the end of rehabilitation. For all HRQOL scales, higher values indicate higher quality of life. Effect size = difference of the mean values divided by the pretest standard deviation.

Before addressing the results of the multivariate analyses, the bivariate correlations between the dependent variables analyzed and all the respective potentially relevant predictors are shown in Table 3. It can be seen that the baseline value always had the greatest correlation with the HRQOL value after inpatient rehabilitation. For the sociodemographic variables, income had the greatest correlation with HRQOL after rehabilitation and for medical variables, the risk score. Both the personality variables and the aspects of the physician-patient relationship correlate significantly with the HRQOL on various scales.

**Table 3:** Bivariate correlations between dependent variables and respective predictors

	SF-12: PC	MacNew: PL	SAQ: PL	SF-12: MC	MacNew: EF	MacNew: SF
**Respective base-line value (at start of rehabilitation)**	.661***	.700***	.707***	.649***	.772***	.700***

						

**Age**	-.220***	-	-	.087	.146*	-

**Gender: man**	.179*	-	-	.188**	.095	-

**Living with a Partner**	-	-	-	-	-	-

**Education: Elementary School**	-.131	-.095	-	-	-.051	-.048

**Employment**	-	-	.274***	-	-	-

**Income**	-	.273***	-	.243***	.222***	.198**

						

**Acute event**	-	-	-	-	-	-

**Myocardial Infarction**	-	-	-	-	-	-

**PTCA**	.291***	-	-	-	-	-

**Bypass**	-	-	-.360***	.016	-	-

**BMI**	-	-	-	-.021	-	-

**Smoker**	.087	-	-	-	-	-

**Risk score**	-.116	-.216***	-.219***	-	-	-.198**

**Total cholesterol**	-	-	.080	-	-.044	-

**LDL cholesterol**	-	-	.115*	-	-	-

**Systolic blood Pressure**	-	-	-	-.025	-	-

**Diastolic blood Pressure**	-	-	-	-	-	-

						

**Cynicism**	-	-.212**	-.186**	-	-	-

**Trait anger**	-	-.231***	-	-.325***	-.377***	-.277***

						

**PICS: DF**	.084	.162**	.109	.080	.151**	.103

**PICS: PI**	-.013	.013	-	-.085	-	-.030

**Trust in Physician**	-	-	-	.208*	.290***	.197***

Pearson correlations with significance level, *** p < .001, ** p < .01, * p < .05, - = not in the model

Table 4 presents the results of the hierarchical regression analyses. The values of the VIF are generally below 2 and always below 5, so there is no indication of high multicollinearity. The baseline values explain between 42.0% and 59.4% of the variance of the HRQOL values after rehabilitation. The sociodemographic variables show significant incremental explanation of variance on all HRQOL scales. The additional explained variance ranges between 0.7% and 5.0%. The most significant sociodemographic variable is - just as for the univariate analyses - income, and the influence of income on emotional and mental aspects of the HRQOL is even clearer. Even taking all confounders mentioned here into consideration, persons with higher incomes also indicate a higher psychological HRQOL at follow-up; i.e., they appear to benefit more from rehabilitation.

**Table 4:** Hierarchical regression analyses to predict HRQOL at the end of rehabilitation

	SF-12: PC	MacNew: PL	SAQ: PL	SF-12: MC	MacNew: EF	MacNew: SF
**Block 1**						

Respective baseline value (at start of rehabilitation)	**.631 **(p < .001)	**.613 **(p < .001)	**.605 **(p < .001)	**.582 **(p < .001)	**.723 **(p < .001)	**.633 **(p < .001)

	R^2 ^change = .436***	R^2 ^change = .488***	R^2 ^change = .498***	R^2 ^change = .420***	R^2 ^change = .594***	R^2 ^change = .488***

**Block 2**						

Age	-.112 (p = .050)	-	-	.090 (p = .105)	**.087 **(p = .049)	-

Gender: man	.083 (p = .105)	-	-	.079 (p = .083)	**-.084 **(p = .029)	-

Living with a partner	-	-	-	-	-	-

Education: elementary school	**-.151 **(p = .002)	-.057 (p = .214)	-	-	-.034 (p = .436)	-.058 (p = .226)

Employment	-	-	.070 (p = .113)	-	-	-

Income	-	.082 (p = .149)	-	**.151 **(p = .013)	**.119 **(p = .004)	.088 (p = .106)

	R^2 ^change = .050***	R^2 ^change = .011*	R^2 ^change = .007*	R^2 ^change = .037*	R^2 ^change = .023**	R^2 ^change = .013*

**Block 3**						

Acute event	-	-	-	-	-	-

Myocardial infarction	-	-	-	-	-	-

PTCA	**.139 **(p = .042)	-	-	-	-	-

Bypass	-	-	**-.139 **(p = .003)	.049 (p = .347)	-	-

BMI	-	-	-	.074 (p = .133)	-	-

Smoker	-.082 (p = .280)	-	-	-	-	-

Risk score	-.055 (p = .350)	**-.113 **(p = .017)	**-.123 **(p = .016)	-	-	-.103 (p = .067)

Total cholesterol	-	-	.114 (p = .392)	-	**.074 **(p = .044)	-

LDL cholesterol	-	-	.028 (p = .746)	-	-	-

Systolic blood pressure	-	-	-	.067 (p = .369)	-	-

Diastolic blood pressure	-	-	-	-	-	-

	R^2 ^change = .024**	R^2 ^change = .004	R^2 ^change = .038**	R^2 ^change = .010	R^2 ^change = .005*	R^2 ^change = .005

**Block 4**						

Cynicism	-	-.077 (p = .219)	**-.108 **(p = .033)	-	-	-

Trait anger	-	**-.095 **(p = .028)	-	-.118 (p = .091)	**-.080 **(p = .032)	**-.144 **(p < .001)

	R^2 ^change = .000	R^2 ^change = .015**	R^2 ^change = .006	R^2 ^change = .013**	R^2 ^change = .006**	R^2 ^change = .020***

	**SF-12: PC**	**MacNew: PL**	**SAQ: PL**	**SF-12: MC**	**MacNew: EF**	**Mac New:SF**

**Block 5**						

PICS: DF	**.130 **(p = .049)	**.220 **(p = < .001)	**.130 **(p = .004)	.099 (p = .357)	.069 (p = .148)	**.132 **(p = .025

PICS: PI	-.127 (p = .187)	-.089 (p = .276)	-.123 (p = .076)	-		-.101 (p = 183

Trust in physician	-	-	-	.095 (p = .086)	**.087 **(p = .046)	.052 (p = .384)

	R^2 ^change = .013*	R^2 ^change = .029***	R^2 ^change = .015**	R^2 ^change = .022**	R^2 ^change = .017**	R^2 ^Change = .016**

						

**R^2^**	**.523**	**.547**	**.563**	**.503**	**.645**	**.542**

Standardized regression coefficients and R^2 ^change with significance, *** p < .001, ** p < .01, * p < .05, - = not in the model. Significant parameters (p < .05) are printed in bold type.

The characteristics of the CHD and the risk factors explain only little incremental variance beyond the sociodemographic variables. The amount ranges between 0.4% and 3.8% and is often not significantly different from zero. The most important predictor variable within this block of variables is the cardiac risk score, which is particularly significant with respect to the physical component of HRQOL. Persons with a higher risk score, that is with a higher probability for death or myocardial infarction, have a lower HRQOL after rehabilitation, i.e. they benefit less from rehabilitation with respect to HRQOL.

On all three psychosocial scales and on the "physical limitation" scale of the MacNew questionnaire, the two personality variables trait anger and cynicism together explain a significant portion of incremental variance, which is albeit not too high (between 0% and 2.0%). For the psychosocial HRQOL, trait anger appears to be more important than cynicism. Persons who tend to be angry in many situations have a lower HRQOL after rehabilitation, even after adjusting for sociodemographic variables, characteristics of the CHD, and risk factors. Cynicism also proves to be a relevant risk factor for one of the HRQOL constructs - the physical limitation measured by the SAQ is less favorable if the person exhibits negative and cynical attitudes.

In the last step, after the baseline HRQOL values and after sociodemographic, medical, and personality variables, aspects of the physician-patient relationship perceived by the patient were included in the regression model. Although there were thus strict conditions for achieving additional explanation of variance, the aspects led to a significant increase in explained variance for all six HRQOL scales. The amount fluctuated between 1.3% and 2.9% and is thus generally greater than the increase in explanation of variance by the characteristics of the disease. The relevance of this block of variables is mainly due to the doctor facilitation scale of the PICS: patients who experienced the physician as a provider who attempted to include the patient in the treatment (e.g. asked the patient for consent; asked the patient what he considered to be the causes of his condition; encouraged the patient to give his opinion), had a higher quality of life at the end of rehabilitation than patients who experienced a patriarchal style of interaction. Trust in the physician was only occasionally a significant predictor in the multivariate analyses; the patient's active communication behavior (patient information scale of the PICS) does not appear to be relevant.

## Discussion

The two hypotheses presented in the introduction were confirmed, at least for some of the sub-dimensions. The relevance of the personality variables was shown in particular for the psychosocial aspects of the HRQOL, while the aspects of the physician-patient relationship are important for all domains of the HRQOL. The confirmation of the significance of anger and cynicism is compatible with existing studies [21-23], and it should be mentioned as a strength of our study that a number of disease characteristics and body function parameters were controlled and that the HRQOL was measured using various instruments. It was seen that the disease characteristics are generally less significant, with the exception of the risk score as a measure of the severity of the illness. While the differences between the various HRQOL scales are considerable for individual predictors, the fundamental relevance of the personality variables and the physician-patient relationship was seen to be independent of whether the SF-12, the MacNew questionnaire, or the SAQ was used.

Our data also show the relevance of income for the improvement of the psychological quality of life. After the baseline value, income was the most important predictor variable for the SF-12-MC as well as the MacNew-EF. Literature reports on socioeconomic status (SES) as a moderator of the correlation between anger and subclinical atherosclerosis for members of the general population (cf. [11]) and occasionally income is also adjusted (cf. [4]), but we are not aware of any study that has examined personality variables and SES in parallel for the prediction of the HRQOL after cardiac rehabilitation. Studies that examine influence factors of the outcome of cardiac rehabilitation independently of personality variables occasionally also indicate the influence of the SES (e.g. [30]), however, there was not always a clear distinction made between education and income (cf. [31]). Our data indicate that low income and low education level are separate risk factors, whereas only income is an influence factor of the psychological HRQOL. Corresponding with this finding, in our sample there is only a slight correlation between the two variables (r = 0.28). The significance of SES for the HRQOL in the context of cardiac rehabilitation is not surprising, although there has thus far been little proof, as social inequality in cardiovascular diseases has been studied on several occasions and correlations have been found, for example with heart disease mortality [51].

In the analysis of the influence of the physician-patient relationship on the HRQOL, the physician's inclusion of the patient in treatment planning proved to be a significant conducive factor for the physical and social HRQOL. This finding made in a prospective study does not reach the level of evidence of a randomized controlled trial, but since an extensive control of potential confounders was made and patient participation was not included until the last block of the model, it does appear to us to be an argument for the importance of patient-oriented communication style and for the relevance of the SDM concept. The results are compatible with intervention studies (e.g. [52,53]) and observation studies [54], which show that for cardiac disease, including the patient in the decision-making process leads to a positive effect on the treatment outcome. However, on the basis of our data we cannot make any statements about the causal path that ultimately leads from the physician's interaction style to an improvement in the HRQOL. A mediator function of therapy adherence is conceivable [55].

It was surprising that active patient communication aimed at participation (e.g. asking physicians to explain treatment in more detail; asking physicians about symptoms of the illness) did not have a significant influence on any HRQOL scale. This was in contrast to studies that show that patient participation can be improved with comparably easy-to-conduct patient communication trainings (cf. [56,57]). It is possible that, without intervention, the influence of proactive patient behavior is too weak to influence the treatment process and thus the HRQOL at the end of treatment. The only slight significance of trust in the physician leads to the assumption that the decisive factor influencing the physician-patient relationship for the cardiac patients studied here is not so much the quality of the emotional relationship as it is the physician's function in activating the patient and emphasizing his own responsibility. The assumption that the meager influence of trust in the physician is based on ceiling effects that are known from patient satisfaction research [58] was not confirmed; the Trust-in-Physician questionnaire has good distribution properties (data not presented in the results section).

The relevance of income for HRQOL presented above underlines the usefulness of the multiple imputation method chosen here. Since a relatively large number of missing values can be anticipated for data on income (cf. [59]; in our dataset, the percentage of missing values was 24.8%) and a selective loss of data can be expected [60], a meaningful analysis of the relevance of income is difficult to achieve without an elaborated imputation method.

Our study has many limitations that should be addressed in closing. The relatively high decliner rate means that the patients studied cannot be considered representative for all inpatient rehabilitation patients with CHD. Since the most important reason for exclusion was refusal to participate, it is difficult to estimate what influence the high decliner rate has on the results reported here. In a comparable study [61], it was found that decliners are sicker and achieve less improvement after rehabilitation. This would presumably mean that in the group of non-participants, if the number of cases were the same, it would be more difficult to prove the relevance of the predictors studied here.

We studied two personality traits important for CHD patients in parallel, but there are other psychological constructs (e.g. depression [62,23] and sense of coherence [21]), that are closely related to anger und cynicism and possibly function as mediators or moderators. Frasure-Smith and Lésperance [63] emphasize that negative emotions (such as anger or depression) are likely to be correlated with an underlying dimension reflecting a general tendency to experience or report negative emotions, which can possibly be described as neuroticism. This means that we cannot be certain that the correlation with HRQOL found for anger and cynicism are actually only the result of these specific constructs or are perhaps also an expression of a general emotional state. If we had included other psychological variables, an approach using structural equation models to represent individual causal paths between the relevant constructs would have been promising.

It can also not be ruled out that there are other relevant predictors of HRQOL after rehabilitation that we did not cover (for example treatment motivation). It is also conceivable that activity limitations of patients at the start of rehabilitation have an influence and we can present them only to a limited extent with the medical variables and the HRQOL baseline values considered here.

With respect to anger, it should also be stressed that we studied only trait anger as a personality variable, not modes of anger expression (anger-in, anger-out, cf. [9]), which are considered aspects of affect regulation. However, this decision appears useful to us, as the patient in the rehabilitation hospital is in a special setting, so results regarding state variables are difficult to generalize.

Inclusion of the patient in the sense of SDM was measured only in relatively generally terms with the PICS scale. Newer conceptual studies (e.g. [64,65]) emphasize that the SDM construct has many factors depending on the definition; they can be only partially measured with the PICS. In addition, the PICS measures only patient perception, not the physician's assessment.

One limitation of the method is that in our analyses, we could not represent the multilevel structure of the data that stems from the fact that several patients were treated by the same physicians, as the allocation of patient to physician was not documented when the data was gathered. This aspect is relevant for the variables of the physician-patient relationship. The patient's assessments are not independent of one another for persons who have the same physician; as a result, the standard error for the linear regression is too small and correlations can be overestimated [66]. The problem is somewhat mitigated by the fact that we did not measure the objectively observable behavior of the physician, but rather the behavior assessed retrospectively by the patient. This judgment depends not only on the physician's behavior, but also on the cognitive system of the patient. The dependence of the judgments related to one and the same physician is thus reduced.

Finally, it should be pointed out that we used only questionnaires to measure the physician-patient relationship and not observational procedures or other qualitative methods. This leads to the problem of the common method bias (cf. e.g. [67]). Correlations between the influence factors observed here and the HRQOL could be overestimated because we used the same method to measure them. However, Doty and Glick [68] showed on the basis of published multitrait-multimethod correlation matrices that the common method bias is rarely high enough to cast a doubt on the central results of research.

## Conclusions

The baseline values explain most of the variance of HRQOL after inpatient cardiac rehabilitation. HRQOL depend also on income, personality traits (especially anger), and patient-participation in treatment. These variables cover a relevant portion of the explainable variance of the HRQOL in our study. The study thus confirms findings that are reported in the existing literature mainly for the health care outcomes mortality and body function parameters. Treatment concepts in rehabilitation should include strategies that ensure the success of the intervention even for risk patients (high degree of experienced anger, low income). For providers, an interaction style is recommended that actively includes the patient in the rehabilitation and thus promotes self-management in dealing with the chronic illness.

## Abbreviations

BMI: body mass index; CHD: coronary heart disease; HRQOL: health-related quality of life; PICS: Perceived Involvement in Care Scales; PTCA: percutaneous transluminal coronary angioplasty; SAQ: Seattle Angina Questionnaire; SDM: shared decison making; SES: socioeconomic status; STAXI: State-Trait Anger Expression Inventory.

## Competing interests

The authors declare that they have no competing interests.

## Authors' contributions

EF analyzed the data and prepared the manuscript. MM assisted in the analysis of the data. Both authors contributed to the conception and design of the study. Both authors read and approved the final manuscript.
